# The Need for Ongoing Antimicrobial Stewardship during the COVID-19 Pandemic and Actionable Recommendations

**DOI:** 10.3390/antibiotics9120904

**Published:** 2020-12-14

**Authors:** Wei Ping Khor, Omotayo Olaoye, Nikki D’Arcy, Eva M. Krockow, Rasha Abdelsalam Elshenawy, Victoria Rutter, Diane Ashiru-Oredope

**Affiliations:** 1 Commonwealth Pharmacists Association, London E1W 1AW, UK; weiping.khor@commonwealthpharmacy.org (W.P.K.); omotayo.olaoye@commonwealthpharmacy.org (O.O.); nikki.darcy@commonwealthpharmacy.org (N.D.); victoria.rutter@commonwealthpharmacy.org (V.R.); 2 Department of Neuroscience, Psychology and Behaviour, University of Leicester, Leicester LE1 7RH, UK; emk12@leicester.ac.uk; 3 FADIC School of Antimicrobial Stewardship, Muirfield Road, Watford WD19 6LN, UK; Rasha.Abdelsalam@fadic.net

**Keywords:** antimicrobial stewardship, COVID-19, pharmacy

## Abstract

The coronavirus disease (COVID-19) pandemic, which has significant impact on global health care delivery, occurs amid the ongoing global health crisis of antimicrobial resistance. Early data demonstrated that bacterial and fungal co-infection with COVID-19 remain low and indiscriminate use of antimicrobials during the pandemic may worsen antimicrobial resistance It is, therefore, essential to maintain the ongoing effort of antimicrobial stewardship activities in all sectors globally.

## 1. Introduction

Coronavirus disease (COVID-19), caused by the novel severe acute respiratory syndrome coronavirus 2 (SARS-CoV-2), has been exerting a significant impact on global health care delivery across both primary and secondary care, since it was first reported in December 2019 [1]. As of 20 September 2020, 30 million people globally have tested positive for COVID-19, of which 3.1% have died [2]. It is critical that normal acute infection management is maintained, and potential COVID-19 complications are anticipated. For the majority of patients, COVID-19 will run an uncomplicated course, hospital admission will not be required, and secondary infection will be uncommon [3].

The current SARS-CoV-2 pandemic occurs amid the already ongoing global health crisis of antimicrobial resistance (AMR). Infections caused by antimicrobial-resistant pathogens are estimated to cause 700,000 deaths each year globally and may complicate the care of COVID-19 patients, potentially leading to increased mortality [4], and result in significant economic burden. Resistant infections have previously been highlighted (pre-pandemic era) as causing economic burden and estimated to cost more than 100 trillion US dollars by 2050 if left unaddressed [4]; these are likely to be even more significant in the current pandemic and the post-pandemic era. At time of writing, it is reported that approximately 5% of COVID-19 patients require admission to intensive care units (ICU) and those with significant co-morbidity may require ventilatory support [5]. ICU admission and mechanical ventilation significantly increase the risk of patients acquiring secondary viral, bacterial and fungal infections [6,7].

One risk during a pandemic is that all resources may be diverted to treating patients infected with the pandemic agent, and other key health care priorities may be overlooked or deprioritised. However, antimicrobial stewardship (AMS) programmes remain essential and are likely more important at a time when needs for healthcare resources may exceed capacity [8]. Inappropriate access to and use of antimicrobials during the current SARS-CoV-2 pandemic may worsen AMR globally. This paper aims to contribute to highlighting the need for ongoing action to tackle the global AMR crisis during the COVID-19 pandemic and the need to uphold and continue the principal of AMS programmes among pharmacy teams. First, it focuses on reviewing the key challenges of optimising infection management and minimising AMR during the pandemic. For this, a literature search was performed by extracting published articles from PubMed with the search terms of “antibiotic stewardship”, “antimicrobial stewardship”, “antimicrobial resistance” and “COVID-19”. Subsequently, the article makes actionable recommendations for clinical practice in the context of COVID-19.

## 2. Key Challenges of Optimising Infection Management and Minimising AMR

### 2.1. Continued Occurrence of Common Infections

Amid the current SARS-CoV-2 pandemic, common infections including e.g., seasonal influenza, bacterial infections, tropical infections or malaria will continue to be present [9]. There is no evidence that these common infections should be managed differently during the pandemic [9]. Local, and/or national primary and secondary care infection management guidelines and AMS principles should continue to be followed. Inappropriate use of antibiotics to treat viral infections and indiscriminate use of broad-spectrum antibiotics may reduce availability and lead to resistance and/or increased *Clostridiodes difficile* infections. Chronic infections such as human immunodeficiency virus (HIV) and tuberculosis remain global health issues and may be heavily impacted by the ongoing pandemic, in which diagnosis and treatment may be delayed, inappropriate or interrupted [10].

As governments across the world are closing down cities and restricting movements to flatten the curve of the pandemic, a large proportion of healthcare resources as well as staff are being diverted to halt the spread of COVID-19 [11]. In addition, there is emerging evidence that healthcare staff that lead on AMS have been asked to prioritise COVID-19 response and management, leading to reduced AMS activities.

### 2.2. Empiric Use of Antimicrobials in Patients with Suspected or Proven COVID-19

Testing for SARS-CoV-2 is currently not widely available globally, and the reverse transcription polymerase chain reaction (RT-PCR) test that is currently the gold-standard diagnostic test has a high false negative rate [12]. In the absence of diagnostic confirmation of SARS-CoV-2 infection, it is important that clinical features are carefully assessed to determine the likely source of infection. However, clinical features of SARS-CoV-2 infection are non-specific and can be indistinguishable from bacterial or influenza pneumonia. Some published initial recommendations were to consider empirical broad-spectrum antibiotics and neuraminidase inhibitors when patients presenting with COVID-19 symptoms are admitted to intensive care units [13]. However, it is important to note that the use of broad-spectrum antibiotics can lead to *Clostridiodes difficile* infection and a rise in AMR.

WHO guidance on the clinical management of COVID-19 suggests that antibiotics should not be prescribed for the prevention or treatment of mild COVID-19; while for suspected or confirmed moderate COVID-19 cases, antibiotic therapy should only be offered if there is clinical suspicion of bacterial infection [14]. However, for patients who have suspected or confirmed severe COVID-19, early empirical antimicrobials can be administered to treat all likely pathogens based on clinical judgement, patient host factors and local epidemiology [14].

Other international guidance, for example, The National Institute for Health and Care Excellence (NICE, London, UK) guidelines from the UK, suggest that antibiotics for the treatment or prevention of pneumonia in community settings should not be offered if SARS-CoV-2 is likely to be the cause or if the symptoms are mild [15]. Similarly, in Africa, the Uganda Ministry of Health guidelines do not advocate the use of antibiotics if the patient only suffers from mild COVID-19 symptoms [16].

In spite of this guidance, the empirical use of antibiotics in hospital settings is likely to increase globally because of the ongoing pandemic. In published case studies from China, it has been shown that 100% of severe and moderate cases were treated empirically with antimicrobials such as moxifloxacin and/or cephalosporin [17]. Indeed, this appears to have been standard practice in many hospitals in China [18].

In one hospital in Wuhan, 95% of admitted patients with suspected SARS-CoV-2 infection received antibiotics [19]. Of 191 patients included in the study, 181 patients received antibiotics, but this was shown to have no effect on survival (*p* = 0.15) while 41 patients received antiviral agents, which also had no effect on survival (*p* = 0.87) [19]. Similarly, in a review paper assessing 9 studies conducted in China and the United States including 806 SARS-CoV-2 positive patients, while some patients were found to develop bacterial or fungal co-infection, the comparatively low proportion (8%) did not justify the reported antimicrobial prescribing rates, which included 72% of patients receiving empirical broad-spectrum antimicrobial therapy [20]. A study conducted in the United Kingdom by Hughes et al. showed that the number of bacterial coinfections is low, occurring in 3.2% (27/836) of cases [21]. No evidence of fungal co-infection during early COVID-19 hospital presentation (0–5 days post-admission) was observed [21]. These findings demonstrate that there are currently limited data to support widespread usage of antimicrobial therapy on COVID-19 patients, and there is a need to develop global and regional antimicrobial policies and strengthen AMS interventions to prevent inappropriate use of antimicrobial therapy during the pandemic.

### 2.3. Falsified and Substandard Antimicrobial Medicines

Another challenge during the pandemic response in many countries is combating falsified and substandard medicines and pharmaceutical supplies. Falsified medicines are medicines which have no or little active ingredients and have not undergone any quality control evaluation, while substandard medicines are authorised medical products that fail to meet either their quality standards or specifications or both [22]. Past studies have shown that falsified or substandard antimicrobials are highly likely to promote the emergence and spread of AMR [23]. Recent studies have also shown that the resistance of *Escherichia coli* and *Mycobacterium smegmatis* to rifampin occurred as a result of exposure to substandard medicines; this presents a potential threat to tuberculosis treatment [24,25]. Over the years, it has been recognised that addressing the problem of substandard or falsified medicines will require the united action of all relevant stakeholders including government bodies, policy makers, regulatory and law enforcement agencies, public health professionals, patients and the general public. Pharmacists play a pivotal role in combating falsified and substandard medicines by working on strengthening supply chain procurement processes to ensure uninterrupted access to safe and effective medicines during the pandemic [26,27,28,29].

### 2.4. Stock Management and Supply Chain of Antimicrobials

Global antimicrobial supply chains are likely to be affected by the pandemic, and the cost may be increased due to travel restrictions or cancellations, therefore risking patients’ lives and potentially contributing to drug resistance [11]. This is particularly challenging in countries that are highly dependent on imported medicines and pharmaceuticals [30]. Hence, there is a need to apply new innovative supply chain management strategies and diverse supply chains to ensure and protect the supply of essential medicines during the COVID-19 pandemic and beyond [31,32].

### 2.5. Healthcare Associated Infections

Whilst data are currently scant and there is no evidence to suggest that patients with COVID-19 are more likely to be infected by multidrug resistant bacteria and fungi, there is increased possibility that healthcare-associated infections will occur in COVID-19 patients with prolonged hospitalisation [33]. A large proportion of patients admitted to hospital due to severe symptoms required mechanical ventilation (75%), in a Seattle study [34]. Patients may be at increased risk of developing hospital-acquired pneumonia (HAP) and ventilator-associated pneumonia (VAP), which are often associated with drug-resistant bacterial strains. In the first documented outbreak of SARS-CoV-2 in Wuhan, China, VAP occurred in 31% of patients requiring mechanical ventilation and was associated with increased mortality [19]. There is evidence to suggest that a large proportion of deaths during the 1918 Influenza pandemic were due to secondary bacterial infection [35]. Secondary fungal infections must also be considered; putative invasive pulmonary aspergillosis was found in almost one third of critically ill COVID-19 patients at a Parisian hospital [6].

## 3. Recommendations for Adaptations of Clinical Practice in the Context of COVID-19

### 3.1. Consider Existing AMS Principles

Adherence to the local, national and international guideline recommendations is vital to prevent over- and inappropriate prescribing of antimicrobials during the pandemic. To support ease of access to antimicrobial prescribing and COVID-19 management guidelines, the Commonwealth Pharmacists Association (London, UK) developed a new repository of resources on COVID-19 prevention and management via the Commonwealth Partnerships for Antimicrobial Stewardship (CwPAMS) app, which is a smartphone app that consists of national antimicrobial prescribing guidelines from Ghana, Tanzania, Uganda and Zambia as well as international guidelines from the World Health Organization and International Pharmaceutical Federation (FIP) (The Hague, The Netherlands) [36]. A repository of useful resources on COVID-19 may be found in the Supplementary Materials File S1.

Despite limited evidence for the effectiveness of AMS interventions in low- and middle-income countries, AMS alongside infection prevention and control (IPC) remain the cornerstone to tackle AMR [37,38]. Appropriate use of antimicrobials may also reduce the economic burden and ensure availability of antimicrobials given the economic crises during the pandemic [38]. National and/or local levels should continue to develop action plans and policies to promote and perform AMS programmes [38,39]. In recent development, the Commonwealth Pharmacists Association published a CwPAMS toolkit, which outlines strategies and projects that a healthcare facility can implement as part of an AMS workplan. This may serve as a guidance especially for resource-limited countries to initiate AMS programmes [40].

Patient education on the appropriate use of antimicrobials is important as there is no evidence that antibiotics can be used for the treatment of viruses, and research/clinical trials on the use of certain antimicrobials in the management of COVID-19 is still ongoing. On 23 April 2020, the Africa Centres for Disease Control released a statement on medications to treat COVID-19. In the statement, the Africa CDC made the following recommendation:

“Physicians should not prescribe, and individuals should not take, chloroquine or hydroxychloroquine to prevent or treat COVID-19 except under clinical trial or monitored emergency use of unregistered and investigational interventions (MEURI) as these drugs can cause neurologic, ophthalmic, cardiac, and other forms of toxicity and Physicians should not prescribe, and individuals should not take, Lopinavir/Ritonavir, Remdesivir or other medications to prevent or treat COVID-19 except under clinical trial or MEURI” [41].

The WHO’s interim guidance on the clinical management of COVID-19 released on 27 May 2020 also makes the same recommendation stating that:

“Chloroquine and hydroxychloroquine (+/− azithromycin); antivirals including but not limited to Lopinavir/ritonavir, remdesivir, umifenovir, favipiravir; Immunomodulators, including but not limited to tocilizumab and Interferon-β-1a, and plasma therapy should not be administered as treatment or prophylaxis for COVID-19, outside of the context of clinical trials” [14].

Key AMS components that can be promoted and practiced amid the current ongoing COVID-19 pandemic are described below. Actionable recommendations are sub-divided into different sectors of care.

#### 3.1.1. Hospital Care

To improve infection management and reduce AMR in hospital patients, the following principles are likely to be vital [14,39]:

- Appropriate microbiological tests by culture or serological tests based on availability should be obtained before the initiation of empirical antibiotic therapy.
- Local infection management guidelines should be promoted. An initial choice of empirical antibiotic treatment should be selected based on local antibiograms, and institutional antimicrobial guidelines should be based upon local antibiogram results.
- Antibiotic treatment should be evaluated daily, and be deescalated or discontinued if clinical markers are not suggestive of bacterial infection.
- If antibiotic treatment is continued, the choice of antibiotic should be guided by microbiological test results.
- Conversion from an intravenous route to an oral route should be performed as soon as possible, as long as the oral route is not compromised, and the patient has shown clinical improvement.
- The duration of antibiotic treatment can be limited to five days for the majority of respiratory indications.
- Careful patient monitoring is necessary for potential drug interactions or toxicity, e.g., QTc prolongation (macrolides and quinolones), cation drug interactions (doxycycline and quinolones) and other drug interactions (macrolides and quinolones).
- Prophylactic use of antibiotics to prevent bacterial pneumonia should not occur.

#### 3.1.2. Community Care/Primary Care

Primary care providers have the responsibility to support and guide patients through managing COVID-19 symptoms, explaining [42]:

- The common symptoms, which are mostly pyrexia, cough and loss of the ability to smell or taste as well as breathlessness and/or delirium, weakness, headache, muscle pain and sore throat in certain individuals.
- Guidelines to be followed by people caring for them in line with their country’s guidance on self-isolation and protection for vulnerable people.
- The possible outcomes of the disease depending on the severity. If the symptoms are mild, they are likely to feel much better within a week.
- The appropriate health authorities to contact in their country/region if their symptoms get worse, for example NHS 111 online in the UK.

### 3.2. Harness the AMS Role of Pharmacists and Their Teams

Pharmacy teams in the community especially, also play a strategic role in ensuring the rational use of medicines and, as a result, are critically placed to help address AMR [43]. Pharmacists, along with other healthcare professionals, are crucial to ensuring that knowledge and evidence are effectively gathered and provided to members of the public. This ensures the judicious use of medications and prevention of stockpiling of medicines, especially the precious commodities of anti-infective drugs. Individual countries should devise and strengthen prescription-monitoring schemes to monitor the safety and efficacy of any off-label drugs being used for COVID-19 management [44].

Community pharmacists’ dual roles as healthcare providers and retailers has also become very apparent during the pandemic [45], emphasizing their indispensable place as providers of safe and effective medicines and medicines information. Pharmacists and pharmacy associations have a vital role in engaging the public and providing education in communities to ensure timely delivery of scientifically proven and reliable information on COVID-19 prevention and management. In line with this, the CPA alongside other pharmacy bodies such as FIP (The Hague, The Netherlands) and the Royal Pharmaceutical Society (London, UK) organised webinars aimed at ensuring that pharmacists across the Commonwealth, worldwide and UK respectively were equipped with knowledge and the right resources to support the COVID-19 response during the peak of the pandemic. The webinars were designed strategically to prevent common occurrences of misinformation and rumours during the pandemic, which can lead to misuse of medicines and a negative impact on public health [46]. This has been highlighted in the cases of chloroquine, a drug which has a long history of being used for malaria treatment, and hydroxychloroquine, which is commonly used for autoimmune disease treatment. These drugs have come into the limelight through the media as potential COVID-19 treatments. There is currently no randomised controlled trial that suggests their efficacy in preventing or treating COVID-19, and indiscriminate promotion and widespread use of chloroquine and hydroxychloroquine have led to drug shortages, self-treatment, fatal overdoses and potential drug resistance [44]. This is of considerable health concern especially in countries, which are endemic with malaria or have poor assess to reliable and accurate health information.

Community pharmacists are well placed to promote AMS and often have the right knowledge, adequate opportunity and inherent dedication required. Despite this, there is limited information on AMS interventions at a community level with the community pharmacist role being less established and harnessed [47]. Similarly, there is a paucity of data on how community pharmacists have applied the principles of AMS to combat AMR in low- and middle-income countries during the COVID-19 pandemic. Community pharmacists can promote AMS in the context of COVID-19 through [47]:

- Promoting the appropriate use of prescribed antimicrobials for treating infections by advising patients on compliance to the dosage regimen, possible adverse effects and any risk of drug interactions.
- Serving as an interface between prescribers and patients; discussing and consulting with prescribers on antibiotic prescriptions to promote adherence to prescribing guidelines and optimal treatment regimens.
- Advocating for an adequate and effective supply chain to ensure continuous medicines supply and prevent drug shortages.
- Providing advice, counseling and support as well as educating patients on Infection Prevention and Control (IPC) practices, AMR and basic hygiene, including hand washing and COVID-19 transmission, nutritional tips during self-quarantine and the best use of over the counter (non-prescription) medications such as pain relief/symptom control medicines, vitamin C, D and zinc, among other vital medications, especially with special populations such as pregnant and elderly patients [48,49].
- Effectively addressing the increased demand for antimicrobials by providing adequate drug information and a literature review on the treatment options for self-limiting illnesses and guidance on when to see a doctor.
- Utilising the media to organise health education and promotion campaigns on the correct use of antimicrobials during the pandemic, including the provision of guidelines for the proper disposal of old/unused antibiotics or expired medicines to maintain safe antibiotic disposal to reduce medicines in the environment.
- Providing prescribers with updates on the use of antibiotics in bacterial co-infections in COVID-19 patients. This is highly important during the current pandemic, as the WHO reports that the use of azithromycin with hydroxychloroquine is highly prevalent although its use is not yet approved outside of COVID-19 clinical trials [14].

Pharmacy teams across all sectors are at the forefront of contributing to the COVID-19 crisis emergency preparedness. A recent study on global contributions by pharmacists during the COVID-19 pandemic in nine countries discussed how pharmacists worked at the frontline of the pandemic to provide care spanning across a broad range of areas including community pharmacies, hospitals, clinics, public health and care homes among other vital areas [50]. It is, therefore, important for pharmacy teams to be equipped with emergency preparedness skills as well as knowledge on prevention measures for COVID-19 whilst carrying out their duties. This includes [51]:

- Insistence on the adherence to IPC guidelines on hand hygiene, respiratory hygiene and the use of medical masks by patients exhibiting respiratory symptoms.
- The right application of contact and droplet precautions when dealing with suspected cases.
- The provision of health education on the early identification of symptoms, necessary precautions to take and right health facilities patients and families should utilise.
- The application of medication therapy management to ensure patients are receiving right medications properly with regards to their clinical conditions, as well as comprehensive medication management.

### 3.3. Address Issues of Falsified and Substandard Antimicrobial Medicines

As professionals charged with the final custody of medicines, pharmacists have a vital role to play in ensuring that the quality and efficacy of medicines is maintained in these settings, especially with impending challenges in drug supply and access to quality medicines as a result of the pandemic. Substandard and falsified medicines pose significant safety, quality and efficacy risks to patients [52]. In the context of falsified medicines, community pharmacists as frontline healthcare workers have specific duties including:

- Educating patients about the risk of obtaining medicines from unknown and unsafe sources such as unlicensed medicine shops online and medicine hawkers.
- Providing proper documentation and creating a feedback system to identify and track adverse drug events associated with the use of falsified or substandard antimicrobials, coupled with advising patients and providers to report on changes in the efficacy of all medicines.
- Advising governments, healthcare organisations and policymakers to design and implement policies to control the production and importation of falsified and substandard medicines, as well as to improve the detection of the same.

### 3.4. Manage Access to Effective Antimicrobials

Preparedness to ensure hospitals do not run out of antibiotics and other critical drugs is key, and guidance for which medicines stocks to increase should be provided. The WHO has recently published a COVID-19 Essential Supplies Forecasting Tool, which provides guidance on essential drugs including antimicrobials and consumables required to treat severe or critically ill patients [53]. Individual countries will need to conduct active surveillance and establish early warning mechanisms to receive alerts whenever a drug shortage is anticipated by evaluating a drug utilisation review especially for antibiotics. This is particularly important for antibiotics, which are commonly used for community-associated bacterial to lower respiratory tract infections, as shortages are expected to increase urgent care consultations and potentially increase hospital admissions. There has been increasing demand and evidence for the incorporation of digital technology as a tool to monitor stock levels, which provides feedback mechanisms that would ensure the continuity of medication supply.

### 3.5. Ensure Effective Infection Prevention and Control (IPC) Practices

IPC and appropriate use of personal protective equipment (PPE) have been well recognised as ways to control AMR [54]. During a pandemic, these measures are critical and should be expanded to contain the spread of the infections (both the spread of SARS-CoV-2 as well as other hospital-acquired infections [54]. Dedicated, trained IPC teams should be in place where possible. In countries where IPC is limited or non-existent, minimum requirements for IPC must be implemented as soon as possible, both at the national and facility level.

The WHO has issued five strategies to prevent or limit the transmission of SARS-CoV-2 in health care settings. It specifies:

- Ensuring triage, early recognition and source control (isolating patients with suspected COVID-19).
- Applying standard IPC precautions for all patients.
- Implementing empiric additional precautions (droplet and contact and, whenever applicable, airborne precautions) for suspected cases of COVID-19.
- Implementing administrative controls.
- Using environmental and engineering controls.

Hand hygiene and respiratory measures are essential, and all health care workers should be aware of and apply the WHO’s 5 Moments for Hand Hygiene approach [54]. Appropriate selection of hand rub is equally important to ensure optimal antimicrobial efficacy [54]. In accordance with the WHO guidance on local production of hand rub formulation, the Commonwealth Pharmacists Association responded to the COVID-19 pandemic by launching a training video to support pharmacy teams in the production of WHO-formula alcohol-based hand sanitisers to further support IPC in hospitals and prevent the spread of infections, including COVID-19 [55,56].

Whilst access to PPE may be limited, IPC teams should, at the earliest opportunity, assess and quantify demand for masks and hand sanitisers. Local manufacturers of PPE should be identified and engaged to ensure supply where possible. Teams should be trained in appropriate use of gloves and masks—how to use, remove and dispose of them. Interventions to minimise the need for PPE include the use of telemedicine to identify COVID-19 cases, physical barriers such as windows at points of patient contact, restriction in access to patients by visitors and non-essential healthcare workers [57]. PPE should only be used where appropriate to minimise shortages. Environmental cleaning and disinfection procedures should be adhered to.

The use of IPC measures must be supported by administrative controls, including the availability of resources, appropriate infrastructure to allow the segregation of infected patients from the uninfected and distancing of healthcare workers and patients, the development of clear IPC policies and the access to laboratory testing.

### 3.6. Advocate for AMS at the Governmental Level (State or Federal)

The harm of AMR has a widespread effect on not only human health but also other critical priorities including the achievement of universal health coverage and sustainable development goals (SDGs). For example, combatting AMR is important for achieving SDG 3 of “good health and well-being”, because the availability of effective antimicrobials is essential for restoring health where an infection is present. Effective antimicrobials also support the prevention of maternal, neonatal and childhood deaths as well as epidemics of communicable diseases such as tuberculosis, HIV and gonorrhea [58]. Other related SDG goals which AMR can impact include SDG 2 “zero hunger”, SDG 8 “decent work and economic growth”, SDG 6 “clean water and sanitation”, SDG 12 “responsible consumption and production” and SDG 17 “partnerships for the goals” [58]. Considering that AMS is the key action to combat AMR, advocacy at the government level is therefore, an important determinant of its success. Identifying gaps is an important initial step for advocacy; for this, assessing the current level of AMS activities using the WHO checklist of essential national/regional and facility core elements for AMS programmes is recommended [59].

National and international advocacy as well as advocacy through civil societies is particularly important in resource-limited areas [60]. Through the effort of governmental and international collaborations to share established strategies, policies and skills; resource-limited countries may benefit from the experience of countries with existing AMS programmes. This can provide a framework to kickstart and expand AMS programmes without unnecessary delays [60].

## 4. Conclusions

The COVID-19 pandemic is a significant and new public health threat, putting tremendous pressure on all healthcare professionals. However, the ongoing global crisis of AMR must not be neglected. We highlight key challenges of infection management including continued occurrence of common infections, empiric use of antibiotics to treat COVID-19 patients, problematic access to effective antimicrobials and hospital-acquired infections. Given these challenges, we advocate that urgent actions are required to continue AMS practices during the pandemic. Specifically, we highlight the need for the reliance on existing principles of AMS across the hospital sector, primary care and community pharmacy. Other recommendations include ensuring access to effective antimicrobials as well as upholding the principles of IPC. Finally, advocacy for AMS must continue at all levels during the current pandemic and in the post-pandemic era.

## Supplementary Materials

The following are available online at https://www.mdpi.com/2079-6382/9/12/904/s1. Supplementary Materials File S1: A repository of useful resources on COVID-19.
