# Supplementary material for: The Need for Ongoing Antimicrobial Stewardship during the COVID-19 Pandemic and Actionable Recommendations
Source: Antibiotics (Basel). 2020 Dec 14;9(12):904. doi: 10.3390/antibiotics9120904 (PMC7764884; doi:10.3390/antibiotics9120904)
Supplement: Supplementary file 1 [file antibiotics-09-00904-s001.pdf]

Perspectives

The Need for Ongoing Antimicrobial Stewardship During the COVID-19 Pandemic and Actionable Recommendations

Wei Ping Khor [1], Omotayo Olaoye [1], Nikki D'Arcy [1], Eva M. Krockow [2], Rasha Abdelsalam Elshenawy [3], Victoria Rutter [1] and Diane Ashiru-Oredope [1,*]

[1] Commonwealth Pharmacists Association, London E1W 1AW, UK; weiping.khor@commonwealthpharmacy.org (W.P.K.); omotayo.olaoye@commonwealthpharmacy.org (O.O.); nikki.darcy@commonwealthpharmacy.org (N.D.); victoria.rutter@commonwealthpharmacy.org (V.R.)

[2] Department of Neuroscience, Psychology and Behaviour, University of Leicester, Leicester LE1 7RH, UK; emk12@leicester.ac.uk (E.M.K.)

[3] FADIC School of Antimicrobial Stewardship, Muirfield Road, WD19 6LN, UK; Rasha.Abdelsalam@fadic.net (R.A.E.)

* Correspondence: diane.ashiru-oredope@commonwealthpharmacy.org

Supplementary materials File S1:

A repository of useful resources on COVID-19

Commonwealth Pharmacists Association (CPA)

- The Commonwealth Pharmacists Association has created a dedicated email address to provide support. Queries or any resources that could be useful to share with the CPA network should be sent to: covid-19@commonwealthpharmacy.org.

- The CPA has developed the CwPAMS smartphone App contains local and international guidances on COVID-19. To start using it, please visit: https://viewer.microguide.global/CPA/CWPAMS or download the app "MicroGuide" from the smartphone app store. To learn more about the app, watch about it on https://youtu.be/MJ7fa_aLgCI.

- The CPA has launched a training video to support local pharmacy team in the production of WHO formula alcohol-based hand sanitiser to further support infection prevention and control in hospitals and prevent the spread of infections, including COVID-19. Link: https://commonwealthpharmacy.org/press-release-how-to-manufacture-alcohol-hand-rub-training-video-launched-to-support-covid-19-response/.

- A repository of resources on COVID-19 management was developed and can be found at the official webpage of CPA. Link: https://commonwealthpharmacy.org/what-we-do/covid-19-resources-2/.

- Commonwealth Partnerships for Antimicrobial Stewardship (CwPAMS) toolkit provide a collation of resources that a healthcare organisation could implement as part of an Antimicrobial Stewardship workplan. Link: https://commonwealthpharmacy.org/wp-content/uploads/2020/11/CwPAMS-Toolkit.pdf

- Subscribe to the CPA newsletter for more information on COVID-19 at https://commonwealthpharmacy.org/news_and_activity/

World Health Organization (WHO)

- WHO has worked with WhatsApp and Facebook to launch a new WHO Health Alert messaging service to increase access to reliable information. To access the service send the word "hi" to the following number on WhatsApp: +41 798 931 892.

- Publications from WHO on country and technical guidance for COVID-19 can be found at: https://www.who.int/emergencies/diseases/novel-coronavirus-2019/technical-guidance-publications. These includes:

- Infection prevention and control
- Risk communication and community management
- Critical preparedness, readiness and response actions for COVID-19
- Advice for the public

- WHO: Guide to local production of WHO-recommended Handrub Formulations. Link: https://www.who.int/gpsc/5may/Guide_to_Local_Production.pdf?ua=1

- *WHO Risk Communication and Community Engagement (RCCE) Action Plan Guidance COVID-19 Preparedness and Response*: This tool is designed to support risk communication, community engagement staff and responders working with national health authorities, and other partners to develop, implement and monitor an effective action plan for communicating effectively with the public, engaging with communities, local partners and other stakeholders to help prepare and protect individuals, families and the public's health during early response to COVID-19. Link: https://www.who.int/publications/i/item/risk-communication-and-community-engagement-(rcce)-action-plan-guidance.

International Pharmaceutical Federation (FIP)

- Coronavirus SARS-CoV-2/ COVID-19 Pandemic: Information and interim guidelines for pharmacists and the pharmacy workforce https://www.fip.org/files/content/priority-areas/coronavirus/Coronavirus-guidance-update-ENGLISH.pdf.

Africa CDC

- Resources on COVID-19 developed by the Africa CDC may be found at: https://africacdc.org/covid-19/covid-19-resources/.

Regional Technical Support at Africa

- Commonwealth Partnerships for Antimicrobial Stewardship: https://commonwealthpharmacy.org/commonwealth-partnerships-for-antimicrobial-stewardship/
- Africa Centres for Disease Control (Africa CDC): https://africacdc.org/contact-us/
- ReAct Africa: https://www.reactgroup.org/contact/
